# Regulation of miRNAs Affects Radiobiological Response of Lung Cancer Stem Cells

**DOI:** 10.1155/2015/851841

**Published:** 2015-03-01

**Authors:** Yan-mei Xu, Xing-yun Liao, Xie-wan Chen, De-zhi Li, Jian-guo Sun, Rong-xia Liao

**Affiliations:** ^1^Cancer Institute of PLA, Xinqiao Hospital, Third Military Medical University, Shapingba District, Chongqing 400037, China; ^2^Medical English Department, College of Basic Medicine, Third Military Medical University, Shapingba District, Chongqing 400038, China

## Abstract

Radiotherapy (RT) is a key therapeutic strategy for lung cancer, the most common cause of cancer-related deaths worldwide, but radioresistance often occurs and leads to failure of RT. It is therefore important to clarify the mechanism underlying radioresistance in lung cancer. Cancer stem cells (CSCs) are considered the fundamental reason for radioresistance. MicroRNAs (miRNAs) have been regarded as important regulatory molecules of CSCs, carcinogenesis, and treatment response of cancers. It is crucial to clarify how regulation of miRNAs affects repair of DNA damage, redistribution, repopulation, reoxygenation, and radiosensitivity (5R) of lung cancer stem cells (LCSCs). A thorough understanding of the regulation of miRNAs affecting 5R of LCSCs has potential impact on identifying novel targets and thus may improve the efficacy of lung cancer radiotherapy.

## 1. Introduction

Lung cancer is the leading cause of cancer-related deaths worldwide, and non-small-cell lung cancer (NSCLC) accounts for approximately 80%–84% of all lung cancers [1]. Despite recent advances in understanding the molecular biology of lung cancer and introduction of new therapeutic agents to lung cancer treatment, only 15.9% of patients could survive for 5 years [2]. According to the latest Guidelines of National Comprehensive Cancer Network (NCCN) [2, 3], thoracic radiotherapy (TRT) is an important treatment for early- and advanced-stage NSCLC, as a radical or palliative therapy. For example, stereotactic body RT (SBRT) allows local tumor control rate to reach 85%–90% in unresectable patients with stage I-II disease [4, 5]. For patients with stage IIIA-IIIB cancer, concurrent chemoradiotherapy (CRT) would be potentially curative. About 60%–70% of all NSCLC patients develop one or more indications for a radical or palliative radiotherapy (RT) during the course of the disease [6]. However, most of radical or palliative RT cannot eradicate tumors, thus leading to relapse of residual tumors. The local recurrence rate for patients with advanced NSCLC who underwent conventionally fractionated radiotherapy (CFRT) (60 Gy/30 F/6 W) is up to 60%–70% in two years despite application of modern equipment and techniques [7]. Therefore, the radiobiological mechanism underlying radioresistance needs to be explored to improve the efficacy of RT.

## 2. Lung Cancer Stem Cells and Radioresistance

Numerous studies indicate that cancer stem cells (CSCs) may be the fundamental reason for carcinogenesis, metastasis, relapse, and chemoradioresistance of cancer [8–10]. Existence of CSCs has been confirmed in hematopoietic malignancies and all kinds of solid tumors [11, 12]. Lung cancer stem cells (LCSCs) have also been reported. These cells are a rare population with capabilities of unlimited self-renewal, multilineage differentiation, floating sphere formation, and evasion from cytotoxic cancer therapies. More than a dozen of cancer centers have identified and isolated LCSCs from lung cancer cell lines or primary lung tumors [13–28]. These studies demonstrate that LCSCs possess heterogeneity with divergent surface markers (Table 1). Heterogeneity of CSCs is another topic, which will not be further discussed here.

Although there is no generally accepted surface marker, LCSCs have been widely explored for radioresistance phenotype. ALDH1^+^ A549 and SK-BR-3 cells have stem-like properties and indication of radioresistance, which has been confirmed by colony formation assay and *γ*H2AX (phosphorylated H2AX, H2AX is a family member of H2A) foci formation assay [48]. LCSCs sorted from lung cancer cell lines (H125, A549, H1299, and H23) show a reduced apoptotic response and increased survival after irradiation (IR) [49]. Another research indicates that IR decreases proliferation, increases apoptosis, and induces mitochondrion damage in main population (MP) cells, but not in side population (SP) cells which contribute to lung tumorigenesis [50]. All these studies demonstrate that LCSCs make crucial contribution to radioresistance. It is therefore significant to elucidate the radiobiological response of LCSCs, which may have translational implications.

## 3. MicroRNAs (miRNAs) Regulate Biological Behaviors of LCSCs

MiRNAs are a class of evolutionarily conserved, endogenous, small, noncoding RNAs about 17–27 nt in length, which result in translational repression and gene silencing, typically by binding to the 3′-untranslated region (3′-UTR) or amino acid coding sequence (CDS) of the complementary mRNA sequence. Until now, 2588 miRNAs have been identified in human beings (June 2014, the miR Base 21.0), which regulate 60% of the whole genome-wide genes [51]. It has been revealed that miRNAs may play important roles in the regulation of carcinogenesis, self-renewal and differentiation of CSCs, and cancer treatment [52].

A lot of miRNAs have been validated to cause unlimited self-renewal of LCSCs. Lung cancer SP cells express let-7 and miR-31 at lower levels than non-SP cells do. These two miRNAs play opposite roles to keep balance between differentiation and quiescence [53]. In other two studies on lung adenocarcinoma, miR-145 significantly inhibits the proliferation of LCSCs and reduces radioresistance by targeting Oct4/Sox2/Fascin1 [29, 30]. In our previous study, we combined paclitaxel with serum-free medium culture (inverse-induction) to enrich CD133^+^CD326^+^ subpopulation, featuring unlimited self-renewal capacity, from A549 cells. Aberrantly expressed miRNAs, such as miR-29ab, miR-183, miR-17-5p, and miR-127-3P, play critical roles in regulating CD133^+^CD326^+^ subpopulation [21]. Therefore, miRNAs could substantially affect the biobehaviors of LCSCs including their radiobiological response by controlling the signaling pathways of LCSCs. Here, we focus on the potential roles of miRNAs in affecting repair of DNA damage, redistribution, repopulation, reoxygenation, and radiosensitivity (5R).

## 4. Repair of DNA Damage

Cancer cell death during RT is mainly due to DNA double-strand breaks (DSBs) induced by IR. As a result, DNA damage response (DDR) is triggered. It initiates the repair of DNA damage by homologous recombination repair (HRR) or nonhomologous end-joining (NHEJ). This response involves multistep processes and multiple molecules, such as DNA-dependent protein kinase (DNA-PK), ataxia telangiectasia-mutated (ATM) kinase, Kruppel-associated protein 1 (KAP1), complementation group D2 (FANCD2), XRCC2, and XRCC4 [10, 49, 54]. It is reported that CSCs have a more efficient DNA repair mechanism [8, 9]. In ALDH1^+^ LCSCs, repair of DSBs is enhanced [48], and expressions of *γ*H2AX, DNA-PK, ATM, KAP1, and FANCD2 are increased [49]. To some degree, IR remains unselective and indiscriminate to eradicate persistent, drug-resistant tumor stem cell pools [55].

MiRNAs have been proved to regulate the expression of important targets in the DDR pathway. In our previous study, radioresistant lung cancer cells demonstrate stem cell-like properties and downregulated miR-18a expression [56]. And miR-18a inhibits the expression of ATM by constitutively binding to its 3′-UTR [31]. In that case, radioresistant lung cancer cells with downregulated miR-18a possess high capacity of DNA repair and HRR, high phosphorylation level, and nuclear foci formation of *γ*H2AX and 53BP1 [31]. Other miRNAs, such as miR-7 [32] and miR-101 [33, 34], could also directly repress either DNA-PK or ATM and radiosensitize lung cancer cells* in vitro* and* in vivo*. In contrast, miR-210 promotes a more efficient repair of DSBs [35]. Thus, miRNAs could regulate DNA repair of LCSCs in multiple ways.

## 5. Redistribution

Tumor cells in different phases of cell cycle display different degrees of radiosensitivity. After IR, the remaining tumor cells less sensitive to radiation often increase their radiosensitivity through redistribution of cell cycle. However, CSCs exhibit defect in the transition of G1/S and G2/M checkpoints during IR. LCSCs show a reduced apoptotic response, increased survival, less pronounced G2 phase arrest, and S/G2-phase block after IR [49].

Several miRNAs are involved in the regulation of cell cycle in LCSCs. MiR-31 and let-7 induce proliferation of lung cancer SP cells by impacting G0/G1 and G1/S phase transitions [53]. MiR-18a abrogates the IR-induced cell cycle arrest and sensitizes cells to IR [31]. MiR-574-5p significantly promotes the cell cycle entry by inhibiting checkpoint suppressor 1 (Ches1) [36]. MiR-193b represses the expressions of cyclins D1 and uPA and significantly decreases proliferation, migration, and invasion capacities of tumor cells [37]. MiR-26a greatly suppresses the enhancer of zeste homolog 2 (EZH2), inhibits cell proliferation, blocks G1/S transition, and induces tumor cell apoptosis [38]. Hence, miRNAs could play crucial roles in regulating redistribution of cell cycle in LCSCs.

## 6. Repopulation

Repopulation of tumor cells is one of the most common radiobiological responses responsible for the failure of IR. The regrowth rate of a tumor after a sublethal dose of radiation exceeds the growth rate of the untreated tumor. The repopulation of treatment-resistant lung cancer cells, usually with stem-like phenotype [12], is observed from an asymmetric type to a symmetric form in cell division that results in two proliferative daughter stem cells [57]. Signaling pathways, such as Notch, Wnt, and Hedgehog (Hh), able to realize the switch from an asymmetric to a symmetric type of cell division, are prominent in LCSCs [58]. TGF-*β*1 exposure also induces epithelial-to-mesenchymal transition, leading to stemness maintenance, tumorigenicity, invasion, and migration in the CD133^+^ A549 subpopulation [59].

Some miRNAs are discovered within these signaling pathways in lung cancer cells. Dozens of miRNAs could control Notch signaling pathway directly or indirectly. Among them, miR-34a, miR-199b-5p, miR-200b, miR-107, miR-181a-1/b-1, miR-210, miR-34c, miR-124a, miR-7, miR-8/200 family, miR-326, and miR-206 are reported in different tumors including lung cancer [60–63]. Furthermore, a cluster of miR-23a/24/27a is found in NSCLC cells as an oncogene, which could downregulate HMGN2 and E-cadherin and be regulated by TGF-*β* or TNF-*α* [39, 40]. MiR-663 also contributes to proliferation of lung cancer cells by regulating TGF-*β*1 signaling pathway [41]. Repopulation is controlled by these miRNAs and their targets so that the radioresistance of LCSCs is maintained.

## 7. Reoxygenation

Reoxygenation during the interfraction intervals is generally believed to be able to improve the efficacy of IR by enhancing tumor radiosensitivity. It would be interesting to know more about the radiobiological response of CSCs at varying oxygenation levels. Under hypoxia, LCSCs elevate angiogenesis by releasing vascular growth factors, stromal-derived factor, and hypoxia-inducible factors (HIF) and yield highly vascularized remodeling in areas of vasculogenic mimicry [16, 64]. LCSCs are protected from radiation by increasing free radical scavengers and decreasing reactive oxygen species (ROS) [65].

Studies report that miRNAs take part in the process of reoxygenation in LCSCs. Under hypoxia, cancer cells expressing miR-210 show a lower mortality rate owing to a decrease in apoptosis, able to grow even at an IR dose of 10 Gy. A further study shows that miR-210 is induced by HIF-1 and stabilizes HIF-1 in turn through a positive regulatory loop [35]. Another study shows that hypoxia induces miR-155 expression. Vice versa, increasing miR-155 protects lung cancer cells from radiotherapy [42]. All these results confirm that miRNAs play roles in reoxygenation of LCSCs.

## 8. Radiosensitivity

Recently, radiosensitivity has been regarded as the 5th R in radiobiological response of tumors [66]. Basically, radiosensitivity is referred to as the sensitivity of tumor tissues. As mentioned above, the sensitivity of tumor tissues is determined by the CSC subpopulation. Since heterogeneity exists in LCSCs, radiosensitivity of CSCs is also controlled by internal molecules and signaling pathways.

In recent researches, many molecules and signaling pathways are found to function in self-renewal and radioresistance of LCSCs, including p53 mutation [67], Kras mutation [43], NF-*κ*B1 activation [45], and senescence inhibition [46]. Studies reveal that miRNAs are usually involved in these signaling pathways. MiR-34 family, effector of p53 activation, significantly reduces cell survival at an IR dose lower than 4 Gy [47]. A regulatory network (let-7-lin28) formed by let-7a and its repressor lin28 keeps the balance of Kras function by attenuating or activating Kras expression [43, 44]. Downregulated miR-9 and let-7g play critical roles in activation of NF-*κ*B1 [45]. Upregulated miR-214 is the essential reason for radioresistance by inhibiting senescence [46].

In addition, some miRNAs play duplicate or multiple roles in regulating radiobiology of LCSCs. For example, miR-210 could stabilize HIF to promote DNA repair [35] and activate Notch signaling pathway in angiogenesis [60]. And some miRNA families (miR-34 family [47], miR-30 family [68], miR-8/200 family [65], and let-7-lin28 family [43, 44]) are involved in radioresistance of LCSCs. These miRNAs, located in the intersection of different signaling pathways, are possible targets for therapeutic strategies to overcome radioresistance of LCSCs.

## 9. Potential Therapeutic Strategies

Since LCSCs are the source of radioresistance of lung cancer, it seems reasonable and promising to cure lung cancer by eliminating this subpopulation. Mounting preclinical data on putative LCSC targets have emerged. For example, LCSCs express c-kit receptor and produce stem cell factor (SCF), and blocking SCF–c-kit signaling by SCF-neutralizing antibodies or by imatinib (Gleevec) is sufficient to abrogate LCSC proliferation and improve antitumor efficacy [69]. Some potential therapeutic strategies are developed from the knowledge of miRNA involvement in the radioresistance mechanisms in LCSCs. Combining an artificial miRNA (amiR) designed to target 3′-UTR of XRCC2 (an HRR factor) or XRCC4 (an NHEJ factor) with an siRNA to target the gene coding region could more efficiently knock down these genes and radiosensitize lung cancer cells to IR-induced killing [70]. Overexpressed miR-18a is used to downregulate ATM expression by targeting its 3′-UTR and to sensitize cells to IR [31]. Thus, based on the 5R radiobiology, interventions should be given at any level of radiobiological response from repair of DNA damage to reoxygenation and radiosensitivity. We could expand predictions for different therapeutic strategies in combination with radiation. On the one hand, miRNA agomir (for underexpressed miRNAs in LCSCs) or antagomir (for overexpressed miRNAs in LCSCs) should be used in combination with IR to eliminate LCSCs [56]. On the other hand, characteristic miRNAs should be used to selectively image LCSCs by fluorescence* in vivo*. We utilize miR-155, an miRNA enriched in lung cancer cells and LCSCs, combined with molecular beacon to image lung cancer cells* in vivo *[71]. Theoretically, the best approach should be able to monitor CSCs accurately in malignant tissues. A higher dose of IR could thus be given to the CSC-enriching areas so that radioresistance would be managed aggressively [72]. It must be emphasized, however, that at the time of writing this review, lots of approaches to modify radiobiology have not been developed yet. And individual patients differ in their tolerance to RT. If findings on miRNAs associated with radiobiological response of LCSCs could be translated into clinical application, it would be possible to improve the efficacy of radiotherapy.

## 10. Conclusions

**Figure 1:**
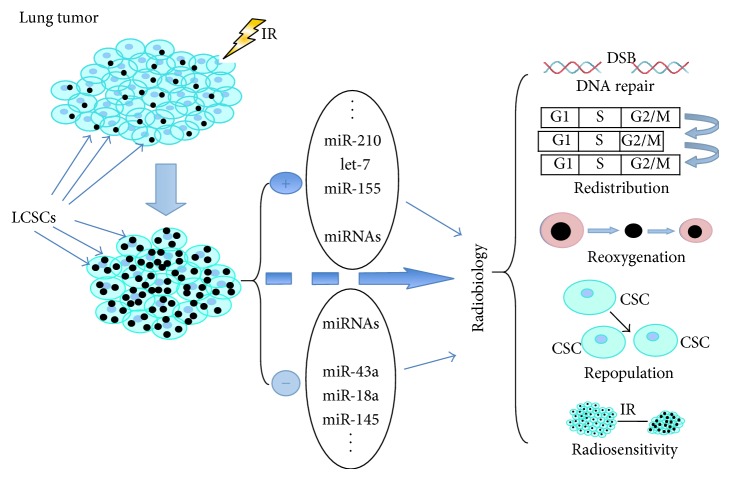
MiRNAs regulate 5R of lung cancer stem cells (LCSCs). After irradiation, lung tumor size is reduced while LCSCs are enriched in the remaining tumors. An aberrant miRNA profile in LCSCs includes over- and underexpressed miRNAs. Thus, DNA repair and repopulation of LCSCs are enhanced, whereas redistribution and reoxygenation are blocked, and radiosensitivity decreases, leading to radioresistance.

By summarizing the principles in modulated radiobiology, we list the important miRNAs and their potential roles in regulating radiobiological response of LCSCs (Table 2). We also draw a diagram on theoretical radiobiology of LCSCs and regulatory miRNAs (Figure 1). Collectively, after IR, the volume of lung tumor decreases, and LCSCs are enriched in the remaining tumors and their percentage increases. The LCSCs not completely sterilized by IR demonstrate an aberrant miRNA profile including overexpressed miRNAs (miR-210, miR-155, let-7, etc.) and underexpressed miRNAs (miR-43a, miR-18a, miR-145, etc.). Therefore, DNA repair and repopulation of LCSCs are enhanced, while redistribution and reoxygenation are blocked, and radiosensitivity decreases, leading to the comprehensive effects of radioresistance.

Accumulating evidence indicates that LCSCs and miRNAs play critical roles in the radiobiological response in lung cancer, and they can effectively control tumor radiosensitivity by affecting pathogenetic cascade of radiobiological effects. Improved understanding of the radiobiology involved in radioresistance offers great promise for developing more effective cancer therapies. Further studies are needed to better understand the mechanism underlying radioresistance and to find a powerful tool to cure lung cancer.

## Figures and Tables

**Table 1:** Biological markers of CSCs in lung cancer.

Biological marker	Source of CSCs	References
Side population	H460	[13]
Side population	H460, H23, HTB-58, A549, H441, and H2170	[14]
Side population	A549, H1650, H460, and H1975	[15]
Side population	A549, H460	[16]
Sca-1^+^/CD34^+^	Primary tumors in mice	[17]
CD133^+^/EP-CAM^+^	Primary tumors	[18]
CD133^+^/CD326^+^	A549	[21]
CD44^+^	10 lung cancer cell lines	[22, 23]
ALDH1A1^+^	Primary lung tumors	[23, 24]
CD166^+^	Primary lung tumors	[25]
CD24(Low/^−^)/CD38^+^	H460	[26]
CD24^+^ITGB4^+^Notch^hi^	Primary lung tumors	[27]
IKKa^low^K5^+^p63^hi^	Primary lung tumors in mice	[28]

**Table 2:** Regulatory miRNAs involved in radiobiological response of LCSCs.

miRNAs	Target genes	Signaling pathway	Radiobiology	Response	References
miR-145	Oct4	Oct4/Sox2/Fascin1	Radiosensitivity	Radiosensitive	[29, 30]
miR-18a	ATM	ATM	Repair of DNA	Radiosensitive	[31]
miR-7	DNA-PK	DNA-PK	Repair of DNA	Radiosensitive	[32]
miR-101	DNA-PK ATM	DNA-PK ATM	Repair of DNA	Radiosensitive	[33, 34]
miR-210	ISCU1/2, etc	HIF-1	Repair of DNA Reoxygenation	Radioresistant	[35]
miR-574-5p	Ches1	TLR9	Redistribution	Radioresistant	[36]
miR-193b	cyclin D1 uPA	G1 arrest	Redistribution	Radiosensitive	[37]
miR-26a	EZH2	G1/S transition	Redistribution	Radiosensitive	[38]
miR-23a/24/27a	HMGN2 E-cadherin	TGF-*β* TNF-*α*	Repopulation	Radioresistant	[39, 40]
miR-663	TGFB1	TGF-*β*	Repopulation	Radioresistant	[41]
miR-155	FOXO3A	Senescence, apoptosis	Reoxygenation	Radioresistant	[42]
let-7a	Kras	Kras	Radiosensitivity	Radioresistant	[43]
lin28	let-7	Kras	Radiosensitivity	Radiosensitive	[43, 44]
let-7g	NF-*κ*B1	NF-*κ*B1	Radiosensitivity	Radioresistant	[44, 45]
miR-214	FoxO4	Senescence	Radiosensitivity	Radioresistant	[46]
miR-34 family	BCL2	p53	Radiosensitivity	Different	[47]

## Abbreviations

5R: Repair of DNA damage, redistribution, repopulation, reoxygenation, and radiosensitivity
CSCs: Cancer stem cells
LCSCs: Lung cancer stem cells
RT: Radiotherapy
TRT: Thoracic radiotherapy
IR: Irradiation
DDR: DNA damage response.
